# Knowledge, attitudes, and practices associated with bioterrorism preparedness in healthcare workers: a systematic review

**DOI:** 10.3389/fpubh.2023.1272738

**Published:** 2023-10-06

**Authors:** Tiantian Li, Yongzhong Zhang, Lulu Yao, Song Bai, Nan Li, Shaotong Ren

**Affiliations:** ^1^Institute of Disaster and Emergency Medicine, Tianjin University, Tianjin, China; ^2^Epidemiology and Health Statistics, Institute of Disaster and Emergency Medicine, Tianjin University, Tianjin, China; ^3^Emergency Medicine, Institute of Disaster and Emergency Medicine, Tianjin University, Tianjin, China; ^4^Evaluation and Optimization of Health Emergency Response Capacity, SD, Institute of Disaster and Emergency Medicine, Tianjin University, Tianjin, China; ^5^School of Management and Economics, Tianjin University, Tianjin, China

**Keywords:** bioterrorism, healthcare workers, knowledge, attitudes, practices, public health

## Abstract

**Introduction:**

Bioterrorism is an important issue in the field of biosecurity, and effectively dealing with bioterrorism has become an urgent task worldwide. Healthcare workers are considered bioterrorism first responders, who shoulder essential responsibilities and must be equipped to deal with bioterrorism. This study aims to extract and summarize the main research components of the bioterrorism knowledge, attitude, and practice dimensions among healthcare workers.

**Method:**

This study utilized a systematic review research design based on the PRISMA 2020 guidelines. A literature search was conducted in the PubMed, Web of Science, and Scopus databases for peer-reviewed literature, and the Mixed Methods Appraisal Tool (MMAT) version 2018 was used to assess the quality of the literature.

**Result:**

A total of 16 studies were included in the final selection. Through the analysis and summary of the included studies, three main aspects and 14 subaspects of the knowledge dimension, three main aspects and 10 subaspects of the attitude dimension, and two main aspects and six subaspects of the practice dimension were extracted.

**Conclusion:**

This study conducted a literature review on bioterrorism knowledge, attitudes, and practices for healthcare workers based on the PRISMA 2020 guidelines. The findings can guide improvements in health literacy and provide beneficial information to professional organizations that need to respond effectively to bioterrorism.

## 1. Introduction

Bioterrorism refers to the intentional release of pathogenic microorganisms such as bacteria, viruses, and toxins, to harm human, animal, and plant health, causing social panic, mass casualties, or serious economic losses (1). Bioterrorism can be executed secretly, results in infection, causes panic, and involves a low cost (1–4). Bioterrorism can not only seriously affect people’s physical and mental health but also lead to the collapse of the public health system and trigger a national crisis (5). Between 1970 and 2019, more than 30 terrorist attacks involving biological agents occurred worldwide, causing widespread international concern (6). The threat of bioterrorism is difficult to eradicate in the short term, and the difficulty of defending against new types of bioterrorism has increased. Thus, effectively dealing with bioterrorism has become an urgent task worldwide.

Healthcare workers are the main force behind bioterrorism preparedness, shouldering essential responsibilities such as surveillance, early warning, and medical treatment, and they are considered bioterrorism first responders. In this process, the emergency literacy of healthcare workers plays a crucial role (7). Emergency literacy is the ability to gain knowledge, acquire skills, maintain a positive attitude and carry out emergency response effectively, and it is closely related to the theory of knowledge, attitude, and practice. The knowledge, attitude, and practice model (KAP model) is one of the classical theoretical models for changing human health behaviors, with layers of progression between the three elements of knowledge, attitude, and practice (8). The current domain of the KAP model regarding emergency response focuses on all hazards (9–11), CBRN events (12–14), and natural disasters (15–18). The research population is focused primarily on professional hospital staff (19–22), and the research indicators are mostly comprehensive assessment indicators (9, 12, 23), but there is a lack of a specific indicator system for bioterrorism. Although existing studies have paid attention to bioterrorism knowledge, attitudes, and practices, they are still inadequately studied. Thus, this review aims to extract and summarize the main research components of the bioterrorism KAP dimensions among healthcare workers by analyzing relevant articles. This study provides beneficial information for professional organizations to respond to bioterrorism effectively and has important implications for improving the emergency literacy of healthcare workers.

## 2. Methods

This study was conducted based on the PRISMA 2020 guidelines (Supplementary materials) (24).

### 2.1. Search strategies

In this study, the PubMed, Web of Science, and Scopus databases were searched for peer-reviewed literature published from January 2008 to January 2023. All database searches included combinations of the following keywords (in the Title/Abstract): “bioterrorism,” “disaster,” “accident,” “event,” “healthcare workers,” “health professionals,” “knowledge,” “attitude,” “practice,” and “preparedness.” The systematic search strategy is shown in Table 1.

**Table 1:** Systematic search strategy.

	Search terms		
	PubMed	Web of Science	Scopus
1. Set of entry criteria	(Bioterrorism) OR (Biological Terrorism) OR (Biological Accident) OR (Biological Event) OR (Biological Disaster) OR (Terrorism, Biological)	TS = (Bioterrorism) OR TS = (Biological Terrorism) OR TS = (Biological event) OR TS = (Biological Accident) OR TS = (Biological Disaster) OR TS = (Terrorism, Biological)	TITLE-ABS-KEY(Bioterrorism) OR TITLE-ABS-KEY(Biological Terrorism) OR TITLE-ABS-KEY(Biological Accident) OR TITLE-ABS-KEY(Biological Event) OR TITLE-ABS-KEY(Biological Disaster) OR TITLE-ABS-KEY(Terrorism, Biological)
2. Set of entry criteria	(Health personnel) OR (Personnel, Health) OR (Health Care Providers) OR (Health Care Provider) OR (Provider, Health Care) OR (Healthcare Providers) OR (Healthcare Provider) OR (Provider, Healthcare) OR (Healthcare Workers) OR (Healthcare Worker) OR (Health Care Professionals) OR (Health Care Professional) OR (Professional, Health Care)	TS = (Health personnel) OR TS = (Personnel, Health) OR TS = (Health Care Providers) OR TS = (Health Care Provider) OR TS = (Provider, Health Care) OR TS = (Healthcare Providers) OR TS = (Healthcare Provider) OR TS = (Provider, Healthcare) OR TS = (Healthcare Workers) OR TS = (Healthcare Worker) OR TS = (Health Care Professionals) OR TS = (Health Care Professional) OR TS = (Professional, Health Care)	TITLE-ABS-KEY(Health personnel) OR TITLE-ABS-KEY(Personnel, Health) OR TITLE-ABS-KEY(Health Care Providers) OR TITLE-ABS-KEY(Health Care Provider) OR TITLE-ABS-KEY(Provider, Health Care) OR TITLE-ABS-KEY(Healthcare Providers) OR TITLE-ABS-KEY(Healthcare Provider) OR TITLE-ABS-KEY(Provider, Healthcare) OR TITLE-ABS-KEY(Healthcare Workers) OR TITLE-ABS-KEY(Healthcare Worker) OR TITLE-ABS-KEY(Health Care Professionals) OR TITLE-ABS-KEY(Health Care Professional) OR TITLE-ABS-KEY(Professional, Health Care)
3. Set of entry criteria	(Knowledge) OR (Attitude) OR (Practice) OR (Preparedness)	TS = (Knowledge) OR TS = (Attitude) OR TS = (Practice) OR TS = (Preparedness)	TITLE-ABS-KEY(Knowledge) OR TITLE-ABS-KEY(Attitude) OR TITLE-ABS-KEY(Practice) OR TITLE-ABS-KEY(Preparedness)
Final Search	1 AND 2 AND 3	1 AND 2 AND 3	1 AND 2 AND 3

### 2.2. Inclusion and exclusion criteria

The inclusion and exclusion criteria used in this study are shown in Table 2.

**Table 2:** Inclusion and exclusion criteria.

Inclusion criteria	Exclusion criteria
1. Articles with content highly relevant to bioterrorism.	1. Articles that only briefly mention bioterrorism.
	2. Articles that do not focus on bioterrorism or that study multiple disasters.
2. Articles with subjects related to healthcare workers (doctors, nurses, dental professionals, paramedics, emergency medical services providers).	3. Articles with research subjects beyond the scope of healthcare workers.
3. Articles that focus on bioterrorism preparedness (including at least one of the three components of knowledge, attitude, and practice).	4. Articles that do not include knowledge, attitude, or practice.
4. Articles in any form (cross-sectional surveys, qualitative studies, literature reviews).	5. Letters to the editor or conference articles with incomplete information.
	6. Articles with missing abstracts or for which the full text cannot be found.
5. Articles published in English.	7. Articles not published in English.
6. Articles published in January 2008–January 2023.	8. Articles published before January 2008 or after January 2023.

### 2.3. Data extraction

The searched article titles were imported into EndNote, which was used to remove duplicate titles. The authors also manually removed duplicate titles by sorting them alphabetically to avoid omissions in software deletion. The article titles and abstracts were screened by two independent reviewers, and disagreements were resolved through negotiation until an agreement was reached. The other two reviewers screened and evaluated full-text articles in the same manner, and promptly recorded the reasons for article exclusion. All reviewers read articles that met the criteria, excerpted scale questions and interview outlines from the articles, and categorized knowledge, attitudes, and practices by brainstorming. The reviewers have been engaged in research investigating health emergency management for a long time, especially research exploring biosafety management.

### 2.4. Quality assessment

This study used Mixed Methods Appraisal Tool (MMAT) version 2018 (25) for quality assessment. The method does not propose that a total score be calculated from the rating of each criterion but rather that a judgment of whether each criterion is met be made. The articles included in this study were independently assessed by two reviewers based on the evaluation checklist and interpretation of the criteria.

## 3. Results

**Figure 1:**
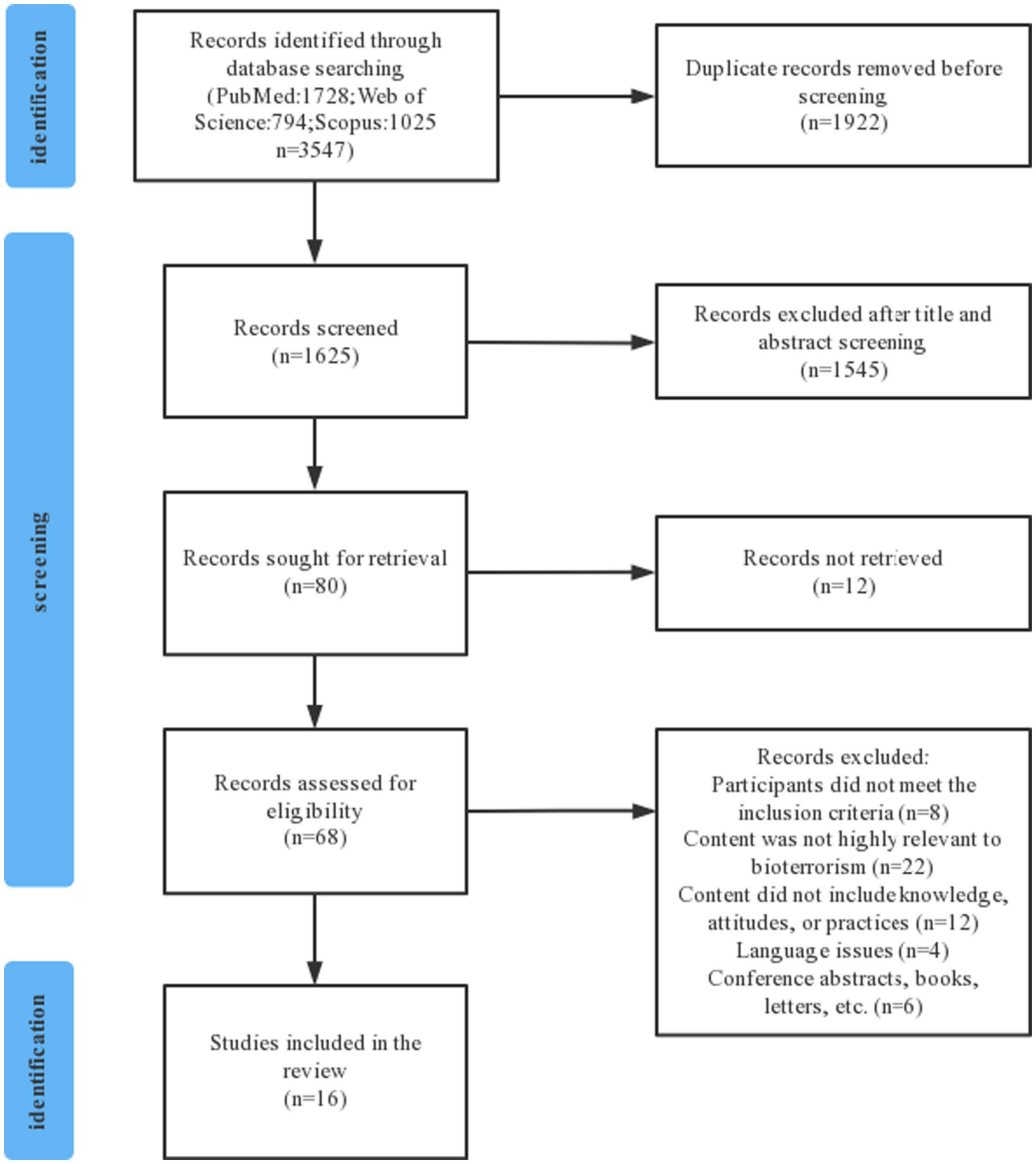
Flow diagram for the selection process.

A total of 3,547 studies were identified in the electronic search, of which 16 were included in the final selection (Figure 1). All included studies were published between 2008 and 2023, and the study subjects included physicians, nurses, dental professionals, pediatricians, and emergency medical services providers. Among the included studies were 1 qualitative study and 15 quantitative studies, and most of the quantitative studies were cross-sectional. Regarding the study region, the vast majority of studies were from the United States (*n* = 9), followed by Iran (*n* = 2), and then Italy, Israel, Ghana, Saudi Arabia, and South Korea, each with one study (*n* = 5). In terms of the study content, 13 studies mentioned the knowledge dimension, 14 mentioned the attitude dimension, and 7 mentioned the practice dimension.

The basic information and MMAT assessment of the final 16 included studies are shown in Table 3.

**Table 3:** Basic information and quality assessment of the included studies.

No.	Author name	Area	Title	Study design/methods	Sample	MMAT	Research dimension
1	Rebmann (26)	America	Missouri Nurses’ Bioterrorism Preparedness	Cross-sectional design	Registered nurses	Criteria 4.2 and 4.4 do not meet the quantitative descriptive study criteria	Attitude, Practice
2	De Felice (27)	Italy	Survey of nursing knowledge on Bioterrorism	Cross-sectional design	Nurses, nursing students	All criteria are met for quantitative descriptive studies	Knowledge
3	Scott (28)	America	Willingness of New England Dental Professionals to Provide Assistance During a Bioterrorism Event	Cross-sectional study	Dental professionals	Criteria 4.2 and 4.4 do not meet the quantitative descriptive study criteria	Knowledge, Attitude, Practice
4	Stankovic (29)	America	Bioterrorism Evaluating the Preparedness of Pediatricians in Michigan	Survey research	Pediatricians	Criteria 4.4 does not meet the quantitative descriptive study	Attitude, Practice
5	Hartwig (30)	America	Critical Challenges Ahead in Bioterrorism Preparedness Training for Clinicians	Survey research	Clinicians	Criteria 4.4 does not meet the quantitative descriptive study	Attitude
6	Crane (31)	America	Assessment of community healthcare providers ability and willingness to respond to emergencies resulting from bioterrorist attacks	Cross-sectional design	Physicians, nurses, pharmacists	All criteria are met for quantitative descriptive studies	Knowledge, Attitude
7	Rokach (32)	Israel	Preparedness for anthrax attack: the effect of knowledge on the willingness to treat patients	Survey research	Physicians and nurses	Criteria 4.4 does not meet the quantitative descriptive study criteria	Knowledge, Attitude
8	Grimes (33)	America	Nurses’ intentions to respond to bioterrorism and other infectious disease emergencies	Descriptive study	Nurses	All criteria are met for quantitative descriptive studies	Knowledge, Attitude
9	Bhoopathi (34)	America	Dental Professionals’ Knowledge and Perceived Need for Education in Bioterrorism Preparedness	Cross-sectional study	Dental professionals	Criteria 4.4 does not meet the quantitative descriptive studies	Knowledge, Attitude, Practice
10	Bork (35)	America	An Assessment of Nurses’ Knowledge of Botulism	Survey research	Registered nurses	Criteria 4.2 does not meet the quantitative descriptive study criteria	Knowledge
11	Aghaei (36)	Iran	Bioterrorism education effect on knowledge and attitudes of nurses	Cross-sectional design	Nurses	All criteria are met for quantitative descriptive studies	Knowledge, Attitude
12	Gorji (37)	Iran	An Assessment of Knowledge and Attitude of Iranian Nurses Toward Bioterrorism	Cross-sectional study	Nurses	Criteria 4.2 does not meet the quantitative descriptive study criteria	Knowledge, Attitude
13	Atakro (38)	Ghana	Nurses’ and Medical Officers’ Knowledge, Attitude, and Preparedness Toward Potential Bioterrorism Attacks	Qualitative explorative descriptive design	Nurses, medical officers	All criteria are met for qualitative research	Knowledge, Attitude, Practice
14	Nofal (39)	Saudi Arabia	Knowledge and preparedness of healthcare providers toward bioterrorism	Cross-sectional design	Physicians, nurses, paramedic/emergency medical services (EMD) team	Criteria 4.2 does not meet the quantitative descriptive study criteria	Knowledge, Attitude, Practice
15	Houser (40)	America	Evaluating Nebraska EMS Providers’ Ability and Willingness to Respond to Emergencies Resulting from Bioterrorist Attacks	Cross-sectional design	Emergency medical services providers	All criteria are met for quantitative descriptive studies	Knowledge, Attitude
16	Lee (41)	Korea	Predictors of bioterrorism preparedness among clinical nurses: A cross-sectional study	Predictive correlational study design	Clinical nurses	All criteria are met for quantitative descriptive studies	Knowledge, Attitude, Practice

### 3.1. Knowledge of bioterrorism

In this study, a total of 16 studies addressed the knowledge dimension of bioterrorism. The authors analyzed and combined these 16 studies, and divided the knowledge dimension into three main aspects, namely, basic knowledge, protection knowledge, and process specification, each of which includes corresponding subaspects. Table 4 shows the main research components of the knowledge dimension.

**Table 4:** Content categories for the bioterrorism knowledge dimension.

Knowledge of bioterrorism	
Main aspects	Subaspects
Basic knowledge	Concepts and nature of bioterrorism (36–38, 41)
	Bioterrorism agent (27, 28, 30, 32–37, 39, 41)
	Early identification (27, 28, 31, 33–35, 39–41)
	Dissemination modes (27, 32, 35–37, 39, 41)
	Epidemiological and clinical characteristics (32, 33, 35, 39, 41)
Protection knowledge	Bioterrorism detection (36, 37)
	Prevention of infection (26, 27, 29, 31–33, 35, 39, 41)
	Control of infection (27, 31–33, 35–37, 39–41)
Process specification	Disease reporting (28, 31, 34, 39, 40)
	Chain of command (31, 39, 40)
	Information communication (28, 31, 34, 40, 41)
	Vaccination (39)
	Decontamination (27, 36, 37, 39)
	Discharge of management control (39)

#### 3.1.1. Basic knowledge

Regarding basic knowledge, bioterrorism agents were mentioned in 11 studies, early identification in 9, and modes of transmission in 7. Moreover, epidemiological and clinical characteristics and the concepts and nature of bioterrorism were mentioned in 5 and 4 studies, respectively.

Bioterrorism agents

Seven studies mentioned six potential category A bioterrorism agents; two targeted single bioterrorism agents, i.e., *Bacillus anthracis* and botulinum toxin; and two posed questions on bioterror agents in measurement tools.

Early identification

Six studies mentioned that common early symptoms were influenza-like illness; two mentioned early symptoms of specific diseases, including foodborne botulism, smallpox, and inhalation anthrax.

Dissemination modes

Aerosol transmission was mentioned in three studies; contact transmission, gastrointestinal transmission, and respiratory transmission were mentioned in two studies; and food-borne transmission and vector-borne organism transmission were mentioned in one study.

#### 3.1.2. Protective knowledge

Regarding protective knowledge, control of infection, prevention of infection, and bioterrorism detection were mentioned in 10, 9, and 2 studies, respectively.

Control of infection

Four studies mentioned negative pressure room isolation, four mentioned patient care and referral, three mentioned patient treatment, and two mentioned symptom monitoring of close contacts.

Prevention of infection

Personal protective equipment was mentioned in five studies, response plans were mentioned in two, vaccination was mentioned in three, and one study assessed anthrax prophylaxis in the measurement tool.

#### 3.1.3. Process specification

In terms of process specification, disease reporting, information communication, decontamination, and chain of command were mentioned in 5, 5, 4, and 3 studies, respectively. Vaccination and discharge of management control were mentioned in 1 study each.

Disease reporting

Five studies mentioned disease reporting procedures, with the three components of reporting scope, reporting timeframe, and reporting subject being the focus of attention.

Information communication

Three studies mentioned access to attack information and clinical information, and two mentioned risk communication, emergency communication devices (phone, fax, radio, satellite phone), and positioning of communication roles in response plans.

Decontamination

Victims and environmental decontamination procedures were mentioned in 3 and 2 studies, respectively.

Chain of command

Two studies mentioned familiarity with the chain of command, and one mentioned who could use the command system for communication during a bioterrorist attack.

### 3.2. Attitudes toward bioterrorism

In this study, a total of 14 studies addressed the attitude dimension of bioterrorism. The authors analyzed and combined these 14 studies, and divided the attitude dimension into three main aspects of cognition, emotion, and behavioral tendency, each of which included corresponding subaspects. Table 5 shows the main research components of the attitude dimension.

**Table 5:** Content categories for the bioterrorism attitude dimension.

Attitudes toward bioterrorism	
Main aspects	Subaspects
Cognition	Perceived risk (26, 28, 29, 34, 41)
	Perceived susceptibility (26, 41)
	Perceived severity (26, 28, 34, 41)
	Perceived benefit (39)
	Perceived barriers (28, 38, 39)
	Perceived competence (28, 30, 34)
	Perceived professional responsibility (28, 31, 33, 34, 39, 40)
Emotion	Psychological feelings (36, 37, 39)
Behavioral tendency	Willingness to respond (28, 31–33, 40, 41)
	Willingness to continue education (28–30, 41)

#### 3.2.1. Cognition

Regarding cognition, perceived responsibility, perceived risk, and perceived severity were mentioned in 6, 5, and 4 studies, respectively. Perceived competence, perceived barriers, perceived susceptibility, and perceived benefit were mentioned in 3, 3, 2, and 1 studies, respectively.

Perceived professional responsibility

Four studies mentioned the suggested role of healthcare workers in emergency response; two mentioned whether bioterrorism preparedness was operational in scope.

Perceived risk

All five studies focused on healthcare workers’ perceptions of the likelihood of bioterrorism.

Perceived severity

All four studies mentioned serious consequences for the state and society, and two mentioned serious effects on the safety and psychological well-being of individuals or the public.

#### 3.2.2. Emotion

All three studies categorized healthcare workers’ psychological feelings as positive, negative, or indifferent.

#### 3.2.3. Behavioral tendency

Regarding behavioral tendency, willingness to respond and willingness to continue education were mentioned in 6, and 4 studies, respectively.

Willingness to respond

All six studies focused on willingness to go to work and provide patient treatment and care during a bioterrorist attack.

Willingness to continue education

All four studies mentioned the willingness to participate in continuing medical education, the preferred method of education, and the recommended length of time.

### 3.3. Practices related to bioterrorism

In this study, a total of 7 studies addressed the bioterrorism practices. The authors analyzed and combined these seven articles, and divided the practice dimension into two main aspects, namely, internal performance and external performance, each of which included corresponding subaspects. Table 6 shows the main research components of the practice dimension.

**Table 6:** Content categories for the bioterrorism practice dimension.

Practices related to bioterrorism	
Main aspects	Subaspects
Internal performance	Participation in continuing education (26, 28, 29, 34, 38, 39, 41)
	Personal response plan (26, 29)
	Family response plan (29)
External performance	Office/workplace response plan (29)
	Guidance of patient response plan (29)
	Guidance of community response plan (29)

#### 3.3.1. Internal performance

Regarding internal performance, participation in continuing education was mentioned in 7 studies, personal response plans in 2, and family response plans in 1.

Participation in continuing education

All seven studies mentioned whether and how many times healthcare workers had participated in bioterrorism-related education.

#### 3.3.2. External performance

In terms of external performance, office/workplace response plans and patient and community response plan guidance were mentioned in one study.

Office/workplace response plan

Office/workplace response plans were mentioned in one study, including coverage of content and content effectiveness.

Guidance of patient and community response plan

One study mentioned the responsibility of healthcare workers to guide patients and communities to develop response plans and to make real-time modifications and refinements.

## 4. Discussion

In this study, a literature review was conducted on bioterrorism knowledge, attitudes, and practices among healthcare workers based on the PRISMA 2020 guidelines. Through the analysis and summary of scale questions and interview outlines from the included studies, a total of 3 main aspects and 14 subaspects of the knowledge dimension, three main aspects and 10 subaspects of the attitude dimension, and two main aspects and six subaspects of the practice dimension were extracted. Regarding knowledge, basic knowledge is the most direct description of bioterrorism, protection knowledge is an important initiative to deal with bioterrorism, and process specification is the provision of standards for bioterrorism management work. Regarding attitudes, cognition is the processing of information about external bioterrorism by an individual, the emotion is the complex and stable physiological evaluation and emotional experience of bioterrorism, and behavioral tendencies are the responses that the individual is prepared to make to bioterrorism preparations. Regarding practices, internal performance is subjects that have a direct relationship with healthcare workers, such as individuals and families, and external performance is subjects that have an indirect relationship with healthcare workers, such as the community and patients.

In this study, only 16 studies were eligible for inclusion through a literature search and screening. This is probably because bioterrorism is an emerging field and research in this area is still in its infancy, with relatively narrow research directions focused on bioterrorism detection technologies (42–44), surveillance and early warning technologies (45–47), and bioterrorism treatment strategies (48–50). Bioterrorism-related studies have been conducted, but studies focusing on bioterrorism KAP are less common and less comprehensive than those on natural disasters.

### 4.1. Knowledge of bioterrorism

Knowledge of bioterrorism can increase the level of response and confidence of health workers (51). This study showed that researchers currently focus on hospital professionals but should also focus on collaboration with first responders from other institutions (52). In addition, current studies mostly focus on groups, and attention should also be paid to the study of individuals in different departments. Insights and roles in bioterrorism work vary between departments; for example, respiratory and dermatology may perform early identification and diagnosis, and neurology may provide treatment decisions (53–55). Finally, Finally, based on past emergency experience, the lack of public understanding of disaster response capabilities makes the medical response system vulnerable to collapse. The public, a non-professional, can be an important complementary force in the necessary moments of disaster response. Public participation should be predicated on raising public awareness of disaster response. Thus, knowledge of the concepts, modes of transmission, and epidemiological characteristics of bioterrorism should be enhanced among public groups to raise public awareness of bioterrorism. It has been argued that the public is important for effective knowledge dissemination, but their understanding of and attitudes toward bioterrorism lack widespread attention (56). A study also pointed out that in addition to national military training, it is important to ensure that a country’s citizens are equipped to effectively respond to bioterrorism (57).

This study found that current research is more focused on bioterrorism agents, early identification, while research on bioterrorism detection is still relatively limited. First, potential bioterrorism agents are internationally classified as A, B, and C (5). Existing studies have focused on category A agents. Although Category A bioterrorism agents have the highest priority, other categories of agents should also receive attention. For example, it was proposed that Category B toxins are readily available and fast-acting, and they are a potential choice for terrorist use in acts of bioterrorism (58). Second, early identification capabilities are crucial to reducing casualties, initiating appropriate treatment, and protecting resources (55). Current research on early identification focuses on the symptoms and signs caused by the disease, and there is a lack of research on epidemiological patterns. For instance, a previous study pointed out that identifying epidemiological patterns, including unusual age distributions of disease, unusual aggregations, and rapidly increasing incidence, is an important task in distinguishing naturally occurring epidemics from terrorist attacks (55). In addition, bioterrorism detection can effectively support clinical diagnosis, surveillance, and epidemiological analysis (42). However, the current research in this area is still relatively sparse, focusing only on the most basic areas of knowledge, and there should also be further research on the operation and application of detection equipment.

Bioterrorism is relatively unique and rare compared to other emergencies, which has led to few opportunities for healthcare workers to learn about bioterrorism. This study found that knowledge does not necessarily contribute to attitudes due to the specificity of bioterrorism. It has been found that there is a positive relationship between the level of knowledge of health workers and their willingness to respond (32, 59). However, other studies have suggested the opposite, namely, that healthcare workers with higher levels of knowledge are less likely to respond positively during bioterrorism (28). Nonetheless, according to the KAP model, knowledge about disease influences health and preventive behaviors (60). Institutions should strengthen bioterrorism training and education to improve the knowledge of health workers.

### 4.2. Attitudes toward bioterrorism

Current research on bioterrorism attitudes has focused on willingness to respond, perceived professional responsibility. Willingness to respond is important in improving hospital surge capacity and maintaining appropriate disaster management. It has been reported that willingness to respond depends on the type of disaster, with healthcare workers more likely to be willing to respond to a natural disaster and substantially less willing to respond to an infectious disease outbreak or bioterrorist event (61). It has also been pointed out that common barriers to the willingness to respond include family and personal response plans, family responsibilities, pet care responsibilities, transportation issues, the need for personal protective equipment. (62).

Surprisingly, perceived professional responsibility plays an important role in bioterrorism preparedness. Healthcare workers are more likely to demonstrate a positive attitude in emergency response due to perceived professional responsibility. It has been reported that physicians who perceive professional responsibility are more than four times more likely to report willingness to respond during outbreaks involving unknown levels of initial risk (63). However, healthcare workers often lack disaster awareness, are unclear about their roles and functions at disaster work sites and are reluctant to respond actively in disaster or relief efforts (64). Thus, healthcare workers’ education on professional responsibility should be strengthened, the number of lectures and trainings should be increased, and appropriate incentive mechanisms should be developed to continuously enhance healthcare workers’ awareness of responsibility, the division of functions, and positive response.

### 4.3. Practices related to bioterrorism

This study showed that participation in continuing education was the focus of attention for the practice dimension of bioterrorism. Response plan development was also of concern, but to a lesser extent.

The emergency response capacity of health workers can be improved through continuing medical education, which also improves the quality of care and increases the effectiveness of teamwork among nurses (64). It has been pointed out that the modalities of continuing medical education include conferences, lectures, seminars, self-study papers, online training scenario simulations. (30). It has also been reported that scenario-based simulations are more effective in enhancing knowledge, preparedness, disaster management, communication, and other areas than traditional education methods such as workshops (51).

Bioterrorism response plans are extremely valuable and relevant as an important safeguard against bioterrorist attacks. It has been reported that healthcare workers in all countries have a responsibility to develop response plans to meet their specific needs and to understand their roles and responsibilities in workplace disaster planning (7). However, current research on response plans is still not thorough enough, and attention should be given to the development of office, community, and patient response plans in addition to research on individual and family response plans.

### 4.4. Limitations

The present study also has some limitations. First, there is relatively little research on bioterrorism and even less focus on bioterrorism knowledge, attitudes, and practices, resulting in a small number of included studies and findings that cannot be generalized to all health workers. In addition, only studies published in English were selected for this study, while potentially relevant studies published in other languages were not selected.

## 5. Conclusion

This study was based on the PRISMA 2020 guidelines and screened studies highly relevant to health workers’ bioterrorism knowledge, attitudes, and practices from January 2008 to January 2023, including both qualitative and quantitative studies. This study analyzed and summarized the key research components of the bioterrorism KAP dimensions based on the included articles and divided them into different aspects and subaspects to explore the role of key components in the bioterrorism preparedness of healthcare workers. The results of this study can improve the emergency literacy of health workers and provide beneficial information for professional organizations seeking to respond effectively to bioterrorism.

Based on the results of the current study, future research could focus on the development of a standardized evaluation framework for knowledge, attitudes, and practices. A stable evaluation framework is the basis for the bioterrorism competency assessment of healthcare workers. Hospital treatment is the end link of the entire disaster response, and future research should also focus on the coordination and linking of medical institutions with the CDC and prehospital emergency care institutions. It is also important to improve the bioterrorism competency of healthcare workers in the CDC and prehospital emergency care institutions.

## Data availability statement

The original contributions presented in the study are included in the article/supplementary material, further inquiries can be directed to the corresponding author.

## Author contributions

TL: Conceptualization, Formal analysis, Methodology, Writing – original draft, Writing – review & editing. YZ: Conceptualization, Funding acquisition, Supervision, Writing – review & editing. LY: Formal analysis, Methodology, Writing – review & editing. SB: Formal analysis, Writing – review & editing. SR: Methodology, Writing – review & editing. NL: Methodology, Writing – review & editing.
